# An improved, time-efficient approach to extract accurate distance restraints for *N*MR^2^ structure calculation

**DOI:** 10.5194/mr-3-137-2022

**Published:** 2022-08-01

**Authors:** Aditya Pokharna, Felix Torres, Harindranath Kadavath, Julien Orts, Roland Riek

**Affiliations:** 1 Laboratory of Physical Chemistry, ETH, Swiss Federal Institute of Technology, HCI F217, Vladimir-Prelog-Weg 2, 8093 Zürich, Switzerland; 2 Department of Pharmaceutical Sciences, Faculty of Life Sciences, University of Vienna, Althanstrasse 14, 2F 353, 1090 Vienna, Austria

## Abstract

Exact nuclear Overhauser enhancement (eNOE) yields highly accurate, ensemble averaged ${}^{1}$ H– ${}^{1}$ H distance restraints with an accuracy of up to 0.1 Å for the multi-state structure determination of proteins as well as for nuclear magnetic resonance molecular replacement ( N MR ${}^{2}$ ) to determine the structure of the protein–ligand interaction site in a time-efficient manner. However, in the latter application, the acquired eNOEs lack the obtainable precision of 0.1 Å because of the asymmetrical nature of the filtered nuclear Overhauser enhancement spectroscopy (NOESY) experiment used in N MR ${}^{2}$ . This error is further propagated to the eNOE equations used to fit and extract the distance restraints.

In this work, a new analysis method is proposed to obtain inter-molecular distance restraints from the filtered NOESY spectrum more accurately and intuitively by dividing the NOE cross peak by the corresponding diagonal peak of the ligand. The method termed diagonal-normalised eNOEs was tested on the data acquired by [Bibr bib1.bibx16] on the complex of PIN1 and a small, weak-binding phenylimidazole fragment. N MR ${}^{2}$ calculations performed using the distances derived from diagonal-normalised eNOEs yielded the right orientation of the fragment in the binding pocket and produced a structure that more closely resembles the benchmark X-ray structure (2XP6) [Bibr bib1.bibx12] with an average heavy-atom root-mean-square deviation (RMSD) of 1.681 Å with respect to it, when compared to the one produced with traditional N MR ${}^{2}$ with an average heavy atom RMSD of 3.628 Å. This is attributed to the higher precision of the evaluated distance restraints.

## 1 Introduction

Nuclear magnetic resonance molecular replacement ( N MR ${}^{2}$ ) is a hybrid approach to determine the structure of protein–ligand complexes, utilising a previously determined structure (for example, a X-ray structure or a structure from a protein homolog) of the target protein and combining it with the spatial information extracted by solution-state NMR to identify the binding pocket of the protein and the orientation of the ligand inside it [Bibr bib1.bibx21]. The major strength of the method is that one does not need to carry out protein resonance assignment to obtain the complex structure. Using N MR ${}^{2}$ , Orts et al. were able to solve the structure of various complexes [Bibr bib1.bibx10] accurately (up to 1 Å) within a few days of measurement and analysis. The N MR ${}^{2}$ structure calculation workflow is detailed in [Bibr bib1.bibx9] and relies on acquiring precise inter-molecular distance restraints.

In N MR ${}^{2}$ , the ${}^{13}$ C, ${}^{15}$ N-labelled protein and non-labelled ligand are mixed and measured together using the F1-[ ${}^{15}$ N, ${}^{13}$ C]-filtered [ ${}^{1}$ H, ${}^{1}$ H] nuclear Overhauser enhancement spectroscopy (NOESY) experiment [Bibr bib1.bibx22] to extract the inter-molecular NOE rates and the corresponding distances. This analysis is performed in an in-built module within CYANA structure calculation software [Bibr bib1.bibx3] called eNORA [Bibr bib1.bibx15]. eNORA fits the NOE build-up curves obtained at multiple mixing times to extract exact cross-relaxation rates (exact nuclear Overhauser enhancements, eNOEs), which produces semi-accurate distance restraints with both upper and lower limit [Bibr bib1.bibx18].

However, the precision of these inter-molecular distance restraints is much lower ( ∼ 20 % higher tolerance needed) [Bibr bib1.bibx14] than the bidirectional intra-molecular eNOEs, usually measured inside the protein, from a series of ${}^{15}$ N, ${}^{13}$ C-resolved [ ${}^{1}$ H, ${}^{1}$ H] NOESY experiments that have a precision of 0.1 Å. The lower precision is attributed to the imbalanced magnetisation pathway within the F1- ${}^{15}$ N, ${}^{13}$ C-filtered [ ${}^{1}$ H, ${}^{1}$ H] NOESY experiment, the lack of a clean steady-state magnetisation at the beginning of the experiment, the unknown spin diffusion contribution [Bibr bib1.bibx4], and the complexity involved in extracting distances within eNORA, which further propagates errors arising from the NOESY spectrum.

In this work, we present an alternative approach for extracting cross-relaxation rates from the filtered 2D NOESY spectra that forgoes the need for the sophisticated and time-intensive eNORA calculations and produces more accurate distances. The complex used in this study is that of cis / trans -isomerase PIN1 with a low-molecular-weight fragment, 2-(3-chlorophenyl)-5-methyl-1H-imidazole-4-carboxylic acid, drawn in Fig. [Fig App1.Ch1.S1.F3], whose structure of the interaction site was solved by [Bibr bib1.bibx16], in order to test the N MR ${}^{2}$ method for weak binding small molecules. This fragment called Compound 2 in the paper by [Bibr bib1.bibx16] produces very few inter-molecular eNOEs to PIN1, due to its small size (comprising only a few protons) and low binding affinity (760 µ M). This makes the de novo determination of the right pose of the ligand in the binding pocket using N MR ${}^{2}$ very challenging.

As we shall see, our approach has been successful in producing better restraints for the PIN1–Compound 2 complex than the standard procedure thereby predicting the right orientation of the ligand in the binding pocket when compared with the X-ray structure of this complex (2XP6) [Bibr bib1.bibx12], which serves as a benchmark to ascertain the accuracy of the N MR ${}^{2}$ structures.

## 2 Theory

Following the standard NMR theory of the NOESY experiment [Bibr bib1.bibx5], the proposed analysis arises out of simple approximations made on the fundamental equations used to calculate eNOEs. Every spin pair that produces a cross peak can be assumed to form a two-spin system. The cross-relaxation rate for a two-spin system ( i and j ) can be analytically given as [Bibr bib1.bibx6]

1 $$\frac{I_{ij}(t)}{I_{ii}(0)}=\frac{I_{ji}(t)}{I_{jj}(0)}=\frac{-\sigma_{ij}}{\lambda_{+}-\lambda_{-}}\left(\mathrm{exp}\{-\lambda_{-}t\}-\mathrm{exp}\{-\lambda_{+}t\}\right),$$

where $I_{ii}$ (t) and $I_{ij}$ (t) represent the peak intensity of the diagonal and the cross peak in the NOESY spectrum respectively. The cross-relaxation rate, $\sigma_{ij}$ , further depends on $\lambda_{\pm }$ , which are a function of auto-relaxation rates of the two spins, $\rho_{i}$ and $\rho_{j}$ .

2 $$\lambda_{\pm }=\frac{\rho_{i}+\rho_{j}}{2}\pm \sqrt{{\left(\frac{\rho_{i}-\rho_{j}}{2}\right)}^{2}+\sigma_{ij}^{2}}$$

The diagonal intensities can be approximated by a single-exponential decay, completely independent of the auto- and cross-relaxation rates of the other spin:

3 $$I_{ii}(t)=I_{ii}(0)\mathrm{exp}\{-\rho_{i}t\}.$$

Furthermore, under the assumption that $\rho_{i}\approx \rho_{j}$ = ρ , which holds true for small- to medium-sized proteins, the exponential terms in Eq. ([Disp-formula Ch1.E1]) can be expanded to the second order as follows:

4 $$\begin{array}{rl} \mathrm{exp}-\lambda_{\pm }t & =\mathrm{exp}\{-(\rho \pm \sigma )t\}\\ & =1-(\rho \pm \sigma )t+\frac{(\rho \pm \sigma {)}^{2}t^{2}}{4}\ldots . \end{array}$$

Combining Eqs. ([Disp-formula Ch1.E1]), ([Disp-formula Ch1.E3]), and ([Disp-formula Ch1.E4]), the following expression can be obtained:

5 .

This straightforward expression relates the cross peak and diagonal intensities at mixing time, t , to the cross-relaxation rate. These quantities can be directly extracted from NOESY spectra recorded at multiple mixing times and fitted with a simple linear model to compute the cross-relaxation rate. This forgoes the need for invoking the eNORA module to fit the NOE build-ups. More importantly, it produces more accurate rates as it only involves directly fitting the experimentally derived peak build-up intensities once. With the standard approach used in eNORA, the diagonal intensities are fitted in accordance with Eq. ([Disp-formula Ch1.E3]) to extrapolate the auto-relaxation rate, $\rho_{i}$ and the initial magnetisation, $I_{ii}(0)$ . The error introduced to these quantities by imprecise fitting of Eq. ([Disp-formula Ch1.E3]) and low signal-to-noise ratio (SNR) of diagonal peaks is propagated to Eqs. ([Disp-formula Ch1.E1]) and ([Disp-formula Ch1.E2]), which are used to determine $\sigma_{ij}$ . Furthermore, the imbalance inherent to the F1-[ ${}^{15}$ N, ${}^{13}$ C]-filtered [ ${}^{1}$ H, ${}^{1}$ H] NOESY experiment and the missing $\rho_{i}$ contributes to the relative error. This error is also compounded in the eNORA approach as the peak intensity data are transformed and used in multiple fitting equations.

The rates determined with the new method proposed here using Eq. ([Disp-formula Ch1.E5]) are termed diagonal-normalised NOEs. The conversion from the obtained cross-relaxation rates to distances can be made via the equations reported in the previous N MR ${}^{2}$ publications [Bibr bib1.bibx21]. Please note that in the case of the PIN1–Compound 2 complex, the effective correlation time used to convert rates to distances was derived from steric distances found in the fragment [Bibr bib1.bibx16].

However, there is a level of uncertainty still attached to the distance restraints extracted via this method because of the assumption, $\rho_{i}=\rho_{j}$ , especially for large ligand–protein complexes with weak binding affinities, as $\rho_{j}$ might be an order of magnitude above $\rho_{i}$ . A simple test was performed to quantify the uncertainty introduced by the above assumption to the extracted distances. It involved taking artificial distances (3 and 5 Å) between spin pairs followed by back-calculating the value of the respective cross-relaxation rates. The obtained rates were fed to Eqs. ([Disp-formula Ch1.E1]) and ([Disp-formula Ch1.E2]) with varying assumptions of the values of the auto-relaxation rates ( $\rho_{j}$ and $\rho_{i}$ ). The ratios of magnetisation transfer $\frac{I_{ij}(t)}{I_{ii}(t)}$ were obtained at identical mixing times [40, 60, 90, and 120 ms], as used by [Bibr bib1.bibx16], and fitted according to the Eq. ([Disp-formula Ch1.E5]) in an attempt to reproduce the artificial distances.

The results of the test are detailed in Fig. [Fig App1.Ch1.S1.F4] in the Appendix. At the ratio of $\frac{\rho_{j}}{\rho_{i}}=10$ , the highest measured ratio generally expected for the complex of a large protein and a small ligand, our method was able to reproduce the inter-molecular distance with an accuracy of 12.45 % for both 3 and 5 Å. Hence, we propose a distance accuracy of ±∼ 10 % for our approach. This distance accuracy lies between distances derived from bidirectional eNOEs (0 %) and unidirectional eNOEs (20 %) [Bibr bib1.bibx14]. It is noted that the CYANA software uses a harmonic potential for a its target function (TF) to accommodate the distance restraints, and as such 0 % means that only harmonic potential is involved, whereas 20 % distance tolerance indicates the presence of a flat potential from 0 %–20 % distance followed by the harmonic potential beyond it [Bibr bib1.bibx3].

## 3 Results and discussion

In order to evaluate the accuracy of the distance extraction method of diagonal-normalised NOEs, the PIN1–Compound 2 complex introduced above is used. All the NMR experiments on the PIN1–Compound 2 complex were conducted and the subsequent resonance assignments were performed by [Bibr bib1.bibx16] (co-authors in this study). They resolved the structure of the binding pocket using N MR ${}^{2}$ on the inter-molecular, unidirectional, eNOE-derived distance restraints which have an expected accuracy of 20 % [Bibr bib1.bibx14]. In this work, we have used their data, recorded on ${}^{15}$ N, ${}^{13}$ C-filtered [ ${}^{1}$ H, ${}^{1}$ H] NOESY spectra, to evaluate the performance of the diagonal-normalised eNOE analysis as compared to the standard eNOE approach [Bibr bib1.bibx18].

**Figure 1 Ch1.F1:**
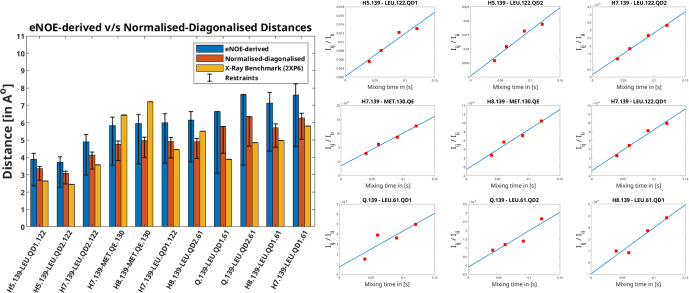
Left: distances extracted from F1-[ ${}^{15}$ N, ${}^{13}$ C]-filtered [ ${}^{1}$ H, ${}^{1}$ H] NOESY using the eNOE (blue) and diagonal-normalised approach (red) compared to the benchmark X-ray structure. The bars denote the distances that arise from the cross-relaxation rates from the complex of PIN1 with Compound 2, as given in [Bibr bib1.bibx16]. The bars in yellow represent the distances back-calculated from the X-ray structure (2XP6) [Bibr bib1.bibx12]. The error bars denote the upper and lower limit restraints produced in CYANA [Bibr bib1.bibx3] for the extracted distances. A tolerance of 20 % and 10 % was taken and for the eNOE and the diagonal-normalised approach extracted distances respectively. Right: the ratio of NOE build-ups to the corresponding diagonal peak intensities plotted against mixing time for the PIN1–Compound 2 complex for the diagonal-normalised approach. The data points were fitted using a linear least square fitting model in MATLAB [Bibr bib1.bibx7]. The slope denotes the cross-relaxation rate of the given peak, as per Eq. ([Disp-formula Ch1.E5]).

Apart from being more time-efficient and intuitive, this method should also provide more accurate distances, as discussed in the Theory section. The NOE build-up plots, fitted linearly according to Eq. ([Disp-formula Ch1.E5]), are depicted in Fig. [Fig Ch1.F1] (right). The linear fits mostly tend to zero when mixing time is zero, and the experimental data fit well, even at longer mixing times for all cross peaks. This indicates a lack of significant spin diffusion contribution. Moreover, it is easier to detect spin diffusion with this method compared to the standard approach using eNORA, as it manifests itself as non-linearity in the fitted data. This difference is illustrated in Fig. [Fig App1.Ch1.S1.F5] in the Appendix.

The derived distance restraints are also plotted against the conventional eNOE-derived distance restraint and the distances back-calculated from the benchmark X-ray structure (2XP6) [Bibr bib1.bibx12] in Fig. [Fig Ch1.F1] (left). (The protons were added to the X-ray structure in CYANA; [Bibr bib1.bibx3].) Indeed, the diagonal-normalised distance restraints better resemble the ones from the X-ray structure (mean difference in the distances being 1.04 ± 0.65 Å) than the ones from the standard approach (mean difference in the distances being 1.57 ± 0.73 Å), the only exceptions being the distances that include the protons from the solvent-exposed Methionine 130.

The inter-molecular distances obtained from the PIN1–Compound 2 complex through the conventional eNORA-based method and the diagonal-normalised approach are plotted in Fig. [Fig Ch1.F1]. The plots illustrate that the restraints obtained via the latter are tighter by 0.4–1.2 Å. The source of this difference, as discussed in the Theory section, arises from the inherent complexity involved in extracting distances from a filtered 2D NOESY spectrum.

**Table 1 Ch1.T1:** Table detailing the results of N MR ${}^{2}$ calculations with distance restraints extracted from eNOE and diagonal-normalised method with varying values of errUni in CYANA.

Method used	Precision	Does the structure	Target function of	Total number	RMSD w.r.t
	(in % of	converge up to	four lowest energy	of degenerate	the benchmark
	the given	TF = 2 Å ${}^{2}$ ?	conformers	lowest energy	(2XP6) (in Å ${}^{b}$ )
	distance) ${}^{a}$	(Yes/No) ${}^{b}$	(in Å ${}^{b}$ )	conformers ${}^{c}$	
eNORA-based	20 %	Yes	[0,0,0,0]	10+	3.63
Diagonal-normalised	20 %	Yes	[0,0,0,0]	5	2.17
Diagonal-normalised	10 %	Yes	[0.03,0.12,0.20,0.73]	1	1.68
Diagonal-normalised	≤ 5 %	No	–	–	–

${}^{a}$ A precision of x % dictates the value of upper limit and lower limit distance restraints with the upper limit distance restraint being ( 1+x %) × (extracted distance) and the lower limit distance restraint being ( 1-x %) × (extracted distance). ${}^{b}$ A target function (TF) of less than 2 Å ${}^{2}$ within N MR ${}^{2}$ is considered a successful structure determination [Bibr bib1.bibx9]. ${}^{c}$ The total number of degenerate lowest energy conformers is the number of distinct orientations of the ligand within the binding pocket that were obtained with a CYANA target function (TF) of 0 Å ${}^{2}$ from N MR ${}^{2}$ calculations.

To evaluate the 10 % error estimate deduced in the Theory section further and to study the impact of the diagonal-normalised distance restraints on N MR ${}^{2}$ structure determination, N MR ${}^{2}$ structures of the complex PIN1–Compound 2 were calculated with varying degree of precision of the diagonal-normalised distance restraints (i.e. 0 %, 5 %, 10 %, and 20 %) (Table 1). The restraints were input in the N MR ${}^{2}$ algorithm, and the output structures were compared to the structure determined in [Bibr bib1.bibx16] using standard eNOEs.

The N MR ${}^{2}$ program screens all potential combinations of methyl groups in protein and protons on the ligand and calculates the complex structure for all of the possibilities without needing protein assignment. The success of an N MR ${}^{2}$ run lies in it being able to discriminate between all the possible structures and pinpoint the right pose of ligand in the binding pocket. This is especially difficult for small fragments like Compound 2, with only five distinct protons/methyl groups.

Table 1 outlines the details of the structure calculation test. The restraints obtained through the eNORA-based method were not good enough and gave rise to more than 10 degenerate structures with a target function (TF) of 0 Å ${}^{2}$ , meaning that all experimental distance restraints were fulfilled without inconsistency/error in any of the 10 degenerate structures. The structure in which the ligand has the same orientation inside the binding pocket as the benchmark X-ray structure (2XP6) [Bibr bib1.bibx12] has a root-mean-square deviation (RMSD) of 3.63 Å with respect to the X-ray structure (2XP6). Using the diagonal-normalised distance determination procedure with a precision of 20 %, a better performance is observed with only five degenerate structures with a TF of 0 Å ${}^{2}$ , which included the complex structure with Compound 2 in the right pose (RMSD of 2.17 Å). For the anticipated precision of the distance restraints of 10 %, the calculation produced only one structure with a TF = 0.03 Å ${}^{2}$ , coloured in purple in Fig. [Fig Ch1.F2], which shows the same orientation as the crystal structure with an RMSD of 1.68 Å. This structure was superimposed onto the benchmark structure, coloured in cyan in Fig. [Fig Ch1.F2]. A visual inspection of the binding pocket illustrated in Fig. [Fig Ch1.F2] shows that the ligand appears deeper in the binding pocket and better aligned with the crystal structure compared to the structure obtained by traditional, eNORA-based N MR ${}^{2}$ . For a distance precision of 5 % and below, the calculations did not converge to structures that fulfil the experimental restraints and produce structures below the hard limit of TF < 20 Å ${}^{2}$ . This is expected since the distance restraints are not of the quality of bidirectional restraints due to the assumption $\rho_{i}=\rho_{j}$ , the lack of spin diffusion correction, and other restrictions inherent to the N MR ${}^{2}$ protocol, such as the use of a previously determined protein structure and the combination of X-ray and NMR data.

**Figure 2 Ch1.F2:**
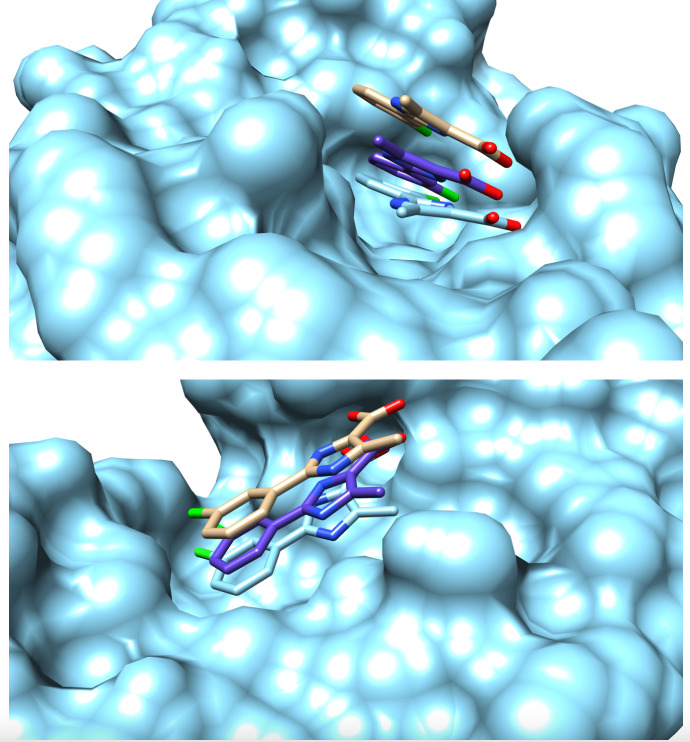
Surface representation of the binding pocket of PIN1 with Compound 2 from two different perspectives. Coloured in cyan is the surface representation of the protein alongside a stick representation of ligand determined by X-ray crystallography studies (2XP6) [Bibr bib1.bibx12]. Coloured in brown is the stick representation of the structure of the ligand inside the binding pocket determined by [Bibr bib1.bibx16] with a distance precision of 20 % using the standard eNORA approach. Coloured in purple is the structure determined by N MR ${}^{2}$ calculations using the distances extracted via the diagonal-normalised approach with a precision of 10 % (refer to Row 3 in Table [Table Ch1.T1]). The nitrogen, oxygen, and chlorine atoms on the ligand are coloured blue, red, and green respectively.

The strength of this approach lies in distinguishing the correct pose of a weak-binding, low-molecular-weight ligand which gives very few inter-molecular NOEs inside the binding pocket of a larger protein. Nevertheless, this approach was also tested on the protein–ligand complex of HDM2, a human oncogenic protein, with caylin-1, which presents abundant inter-molecular NOEs. The traditional eNORA-based N MR ${}^{2}$ was successful in characterising the structure of protein–ligand interaction site (7QDQ), as shown in the work of [Bibr bib1.bibx8]. With the diagonal-normalised approach at 10 % precision, we obtained the same pose of caylin-1 in the HDM2 binding site as [Bibr bib1.bibx8], with a TF of 1.52 Å ${}^{2}$ and RMSD between the two structures of 0.81 Å (refer to Fig. [Fig App1.Ch1.S1.F6] in the Appendix). Furthermore, the calculations made with 15 % and 20 % precision also matched the predictions of traditional N MR ${}^{2}$ in identifying the right structure. This is further evidence that our approach can at least match the predictions of traditional N MR ${}^{2}$ in the case of strong binders and possibly exceed them in the case of weak binders with less NOEs. It is noted that the presence of multiple configurations/conformations of the ligand in the binding pocket will require detailed eNOE-based multi-state structure calculations [Bibr bib1.bibx20].

To sum up, this work proposes an intuitive and time-efficient, alternative method to extract precise distance restraints from a series of filtered NOESY spectra, that gives, in the system studied, an accurate N MR ${}^{2}$ structure of the protein–ligand interaction site.

## 4 Materials and method

No new material was prepared for the sake of this work. The protocol to express and purify the protein and the ligand and to mix them afterwards is detailed in [Bibr bib1.bibx16].

No new NMR experiments were conducted for this work either. The peak intensities from the spectra acquired by [Bibr bib1.bibx16] were extracted via ccpNMR [Bibr bib1.bibx13]. The intensities were later fitted to acquire the rates in the MATLAB software suite [Bibr bib1.bibx7]. The structure calculations were performed by the N MR ${}^{2}$ program through CYANA [Bibr bib1.bibx3]. All the structures were displayed and overlaid each other using the Chimera molecular visualisation tool [Bibr bib1.bibx11].

## Figures and Tables

**Figure A1 App1.Ch1.S1.F3:**
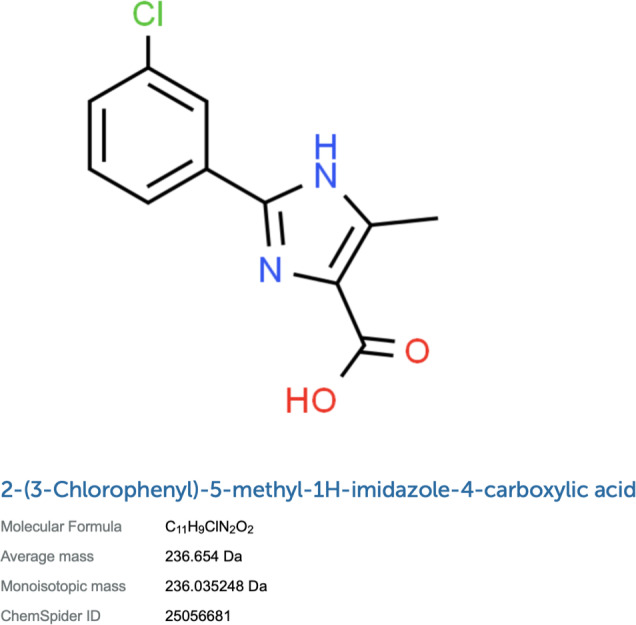
A line representation of Compound 2 (2-(3-chlorophenyl)-5-methyl-1H-imidazole-4-carboxylic acid) from [Bibr bib1.bibx16]. The figure was obtained from ChemSpider (ID:25056681).

**Figure A2 App1.Ch1.S1.F4:**
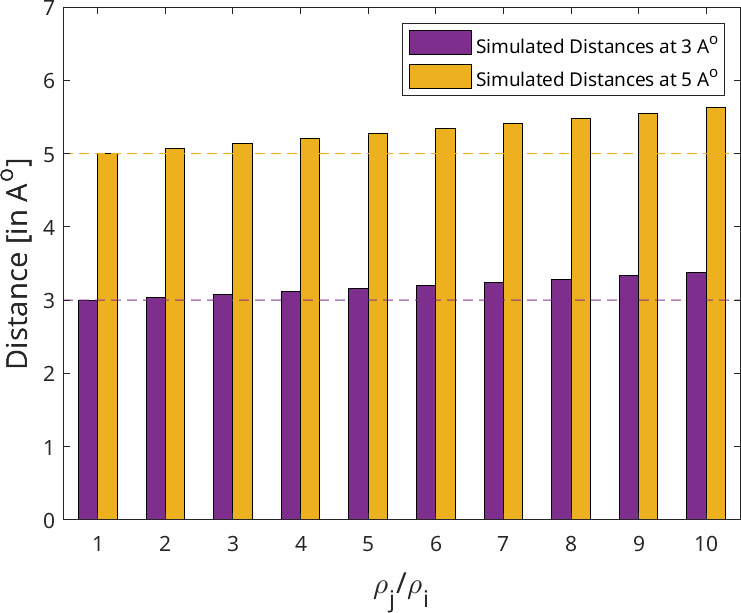
The effect of the relative auto-relaxation rates of the protein and the ligand on the distances extracted via the diagonal-normalised approach. The dotted lines represent the artificial distances 3 and 5 Å in purple and yellow respectively. The corresponding bars denote the distances back-calculated using the diagonal-normalised approach from the artificial distances depending on the relative auto-relaxation rates, $\rho_{i}$ and $\rho_{j}$ . Each set of distances (bars) are derived through varying assumptions of the values of $\rho_{i}$ and $\rho_{j}$ with respect to each other ranging from $\frac{\rho_{j}}{\rho_{i}}$ = [1 to 10].

**Figure A3 App1.Ch1.S1.F5:**
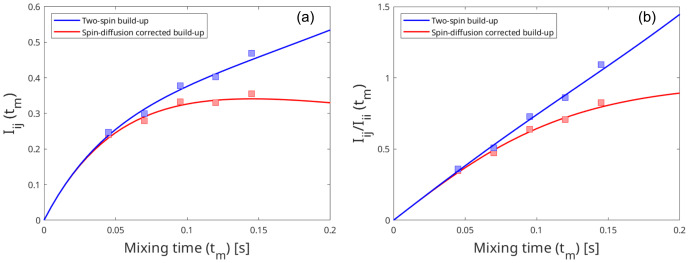
Effect of spin diffusion on the intensity build-up curves produced by the eNORA-based approach **(a)** and the diagonal-normalised approach **(b)**. The build-up curves were fitted using artificially simulated peak intensities in a model system, third immunoglobulin-binding domain of protein G (GB3), which has been extensively studied using eNOE spectroscopy [Bibr bib1.bibx19]. The blue curve represents the intensity build-up in an isolated two-spin system, with an inter-proton distance of 3.83 Å, and the red curve represents the same two spins experiencing spin diffusion due to the presence of other spins in the system. The comparison between the plots highlight that it is easier to detect the influence of spin diffusion with the diagonal-normalised approach **(b)**, as it induces deviation from the expected linear fit.

**Figure A4 App1.Ch1.S1.F6:**
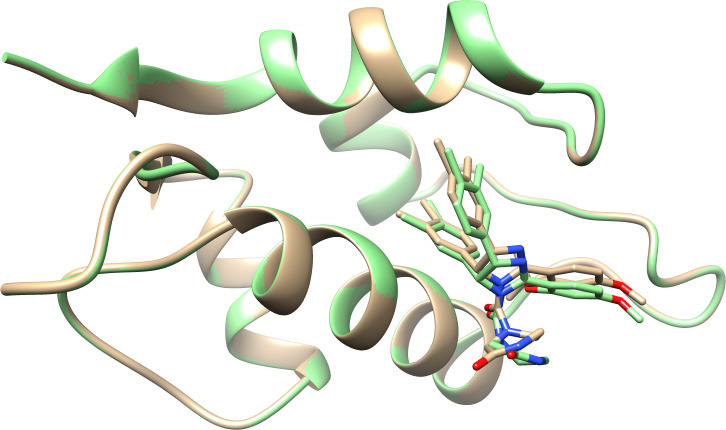
Ribbon representation of the protein, HDM2, with the stick representation of caylin-1 present in the binding pocket. Coloured in brown is the surface of the structure determined by traditional N MR ${}^{2}$ (7QDQ) by [Bibr bib1.bibx8]. Coloured in green is the structure determined by N MR ${}^{2}$ calculations using the diagonal-normalised approach with a precision of 10 %. The nitrogen and oxygen atoms on the ligand are coloured blue and red respectively.

## Data Availability

All relevant data and analysis scripts can be obtained upon request.
